# Jaundice Eye Color Index (JECI): quantifying the yellowness of the sclera in jaundiced neonates with digital photography

**DOI:** 10.1364/BOE.10.001250

**Published:** 2019-02-14

**Authors:** Terence S. Leung, Felix Outlaw, Lindsay W. MacDonald, Judith Meek

**Affiliations:** 1 Department of Medical Physics and Biomedical Engineering, University College London, UK; 2 Department of Civil, Environmental & Geomatic Engineering, University College London, UK; 3 The Neonatal Care Unit, Elizabeth Garrett Anderson Wing, University College London Hospitals Trust, UK

## Abstract

The sclera is arguably a better site than the skin to measure jaundice–especially in dark-skinned patients–since it is free of skin pigment (melanin), a major confounding factor. This work aims to show how the yellowness of the sclera can be quantified by digital photography in color spaces including the native RGB and CIE XYZ. We also introduce a new color metric we call “Jaundice Eye Color Index” (JECI) which allows the yellowness of jaundiced sclerae to be predicted for a specific total serum bilirubin level in the neonatal population.

## 1. Introduction

Neonatal jaundice is a common and sometimes life-threatening condition in the newborn, affecting over 60% of newborn infants [1]. There is an urgent need for cheap, point of care tests to measure jaundice, especially in low and middle-income countries. Jaundice is caused by the accumulation of bilirubin, a breakdown product of red blood cells, in the blood. The yellow-colored bilirubin gives rise to the characteristic yellow discoloration of the skin of jaundiced patients. Since neonatal jaundice usually develops during the first few weeks after birth when the babies have been sent home, the identification of severe jaundice is often carried out without any specialist equipment and by parents or visiting midwives, who tend to perform visual assessment based on the “yellowness” of the skin.

A systematic investigation of the reliability of such visual assessment was carried out by Riskin et al. (2008) involving 5 neonatologists and 17 nurses who visually estimated the bilirubin levels of 1,129 term and preterm infants [2]. They found that although there was a good correlation between the visually-estimated and measured total serum bilirubin (TSB) levels (Pearson’s r = 0.752, p < 0.0001), visual assessment was in fact unreliable as a screening tool to detect significant neonatal hyperbilirubinemia. Babies with high TSB levels might be clinically misdiagnosed as low-risk.

Another way to visually assess the severity of jaundice is by observing the cephalocaudal progression, the spread of jaundiced (yellow) skin throughout the body. Kramer first introduced a grading system to quantify neonatal jaundice based on visual assessment of the skin, using grades between 0 and 5 to describe the extent of jaundice progression [3]. This approach assumes that the occurrence of jaundiced skin patches, or dermal icterus, starts from the head and spreads to the hands and feet as jaundice becomes more severe. It relies on the assessor to determine if a skin region is jaundiced or not - a binary decision, and provide a maximum extent grade (0 - 5) to quantify the severity of neonatal jaundice.

However, when Keren et al. (2009) conducted a study involving 522 term and late preterm babies using this approach, they found that Kramer’s 5-point scale only had a moderate correlation with the measured TSB level, i.e., Spearman’s rho = 0.45 and 0.55 (p = 0.13) for black and non-black babies, respectively [4]. One difficulty with visual assessment of skin color is the presence of melanin which often obscures the yellowness of the skin.

The sclera and the overlaying conjunctiva, on the other hand, are free of melanin and have a high affinity for bilirubin. Therefore, they could be better sites for visually assessing the severity of jaundice. Azzuqa and Watchko (2015) carried out a study involving 240 newborn babies whose eyes were examined by two experienced neonatologists to determine whether conjunctival icterus was present (a binary decision) [5]. They found that visible conjunctival icterus, more often than not, indicated a TSB higher than 255 μmol/L, showing the subjective perception of yellowness in the conjunctiva can identify severely jaundiced newborn babies. Although they also pointed out “conjunctival icterus” as a more appropriate term, we will adopt the more frequently used term sclera to describe the white part of the eye here with the understanding that it incorporates both sclera and its overlying conjunctiva.

The aim of this paper is to demonstrate how the yellowness of sclerae in jaundiced newborns can be quantified in color spaces including the native RGB and CIE XYZ, and to discuss the general requirements of successful color metrics for measuring jaundice. We also introduce a new color metric we call “Jaundice Eye Color Index” (JECI) which allows the yellowness of jaundiced sclera to be predicted for a specific TSB level.

## 2. Methods

### 2.1 Clinical data collection

Eighty-seven newborn babies were recruited in the outpatient clinic on the Neonatal Unit of UCL Hospital. The postnatal age was between 1 day and 28 days. Their TSB were between 17 and 304 µmol/L. A Nikon D3200 digital camera, equipped with a macro lens (60 mm focal length), was used to take digital images of babies’ eyes before they underwent blood tests for TSB as part of their standard care. All images were taken in the same lighting condition and saved in the raw format (NEF, Nikon Electron Format) to avoid any post-processing. The study was approved by National Research Ethics Service Committee (London – City Road and Hampstead), and was conducted according to the Declaration of Helsinki. All parents gave informed consents for their babies to enter the study.

### 2.2 RGB color space

Figure 1(a)

**Fig. 1:**
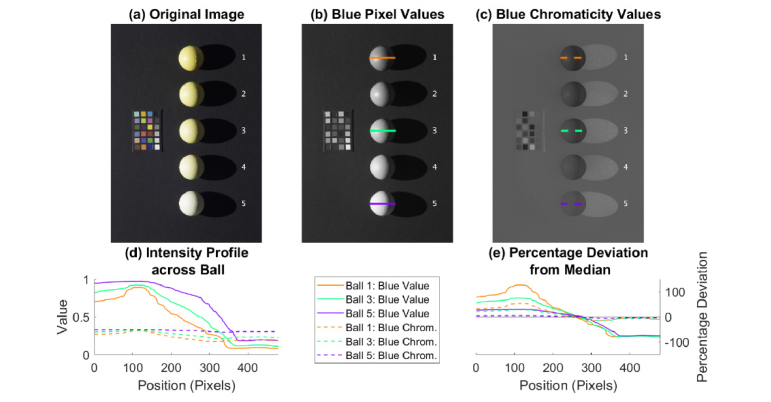
(a) Original image in RGB. Grayscale images of (b) blue pixel values, and (c) blue chromaticity values. The intensity profiles in (d) show that blue pixel values (solid lines) vary over a much greater range for a given ball than blue chromaticity values (dashed lines). The difference can also be seen in (e), which profiles the percentage deviation from each ball median.

depicts a digital image showing 4 balls painted in varying yellowness and 1 white ball. Everyday lighting conditions often give rise to specular reflection and shading which can affect the quantification of color in a digital image. To illustrate this, Fig. 1(b) depicts the blue pixel value image of the 5 balls, parts of which were in shadow. Of the three primary colors, blue pixel values provide the highest contrast of yellowness. In the absence of specular reflection and shading, the blue pixel value profile of each of the five balls should have no variation at all. Figure 1(c) shows the same image in blue chromaticity, defined as the blue pixel value divided by the summation of the red, green and blue pixel values. In Fig. 1(d), the profiles of the blue pixel values (solid lines) across balls 1, 3 and 5 indicate a large variation between 0.1 and 1.0. Figure 1(e) displays the same results in terms of the percentage deviated from the median along the profile, which can be as high as 120% for the specular reflection in ball 1, and as low as −75% for the shadow in balls 1, 3 and 5.

By comparison, the blue chromaticity profiles (dashed lines) of balls 1, 3 and 5 have much smaller variation, as can be seen in Fig. 1(d) and Fig. 1(e), which also shows that shadowing (<10% deviation from the median) has less influence than specular reflection (>30% deviation from the median) on the blue chromaticity. It is evident that chromaticity has the ability to minimize the effect of varying reflectance caused by specular reflection and shading. However, chromaticity still cannot correct for the spectral influence of the ambient lighting on a digital image. Therefore, the most straightforward way to compare color or pixel values of different objects is to do so under the same lighting condition. For analysis purpose, the raw images of babies’ eyes were converted into the standard TIFF format using the open-source program DCRAW (version 9.27 by Dave Coffin 2016) and Matlab (MathWorks, Inc.) was used to analyze the TIFF images. The sclera of each baby was visually identified on the image and the median RGB pixel values were calculated over a region of interest, manually drawn to include the largest continuous area of sclera possible whereby the only criteria were to avoid obvious specular reflection, blood vessels, and eye lashes. The blue chromaticity was also calculated.

### 2.3 CIE XYZ color space

**Fig. 2:**
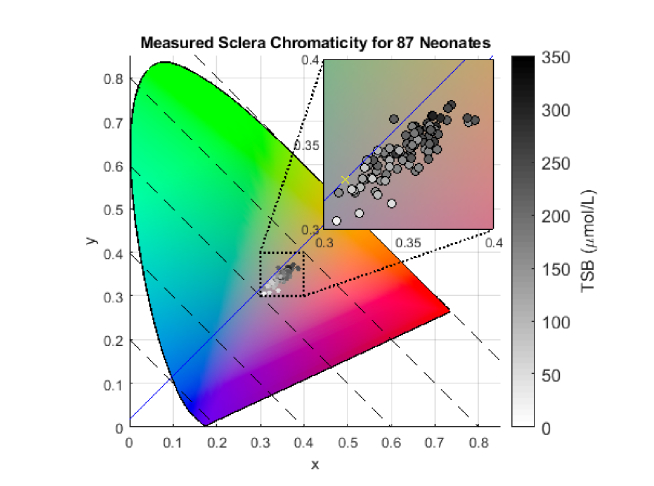
Sclera colors for 87 newborns plotted on the CIE XYZ chromaticity diagram. The blue line is the axis for the Jaundice Eye Color Index. The TSB of each newborn is color-coded.

Human color perception can be quantified using the CIE XYZ color space, which contains all the colors visible to human eyes [6] and is device-independent, unlike the native RGB space. A white patch was identified from the color checker placed near to the baby’s face in each image so that the inverse of the corresponding RGB values could be used as the multipliers for white balance, carried out by the software DCRAW in Matlab. This software also converted the raw image of a baby’s eye into the CIE XYZ space under the D65 illuminant, where the sclera pixels were identified (same procedure as described in section 2.2) and the sclera color was mapped onto the CIE XYZ chromaticity diagram (CIE xy). Each of the 87 data points on the CIE xy diagram shown in Fig. 2

represents the sclera color of a baby. We define a new color metric known as Jaundice Eye Color Index (JECI):

$$\text{JECI}=z_{D65}-z=\left(z_{D65}-1\right)+x+y$$ (1)

where z_D65_ = 1 – 0.313 – 0.329 = 0.358 for D65 illuminant. The D65 white point, marked by a yellow cross in Fig. 2, is located at (0.313, 0.329). JECI can be considered as the orthogonal projection of a data point onto the diagonal axis y = x + m (blue diagonal line on Fig. 2). For D65 illuminant, m = y_D65_ - x_D65_ = 0.329 - 0.313 = 0.016. Along each of the dashed diagonal lines (y = offset - x), which are all perpendicular to the line y = x + 0.016, the values of JECI are the same. JECI is essentially z chromaticity with the constraint of a zero value at the D65 white point. It is sensitive to an object’s yellowness as the $\bar{z}$ color matching function overlaps with the blue region of the visible spectrum (where bilirubin absorbs strongly) [6]. The 87 data points in Fig. 2 are color-coded with darker colors corresponding to higher TSB values.

## 3. Results

### 3.1 RGB color space

**Fig. 3:**
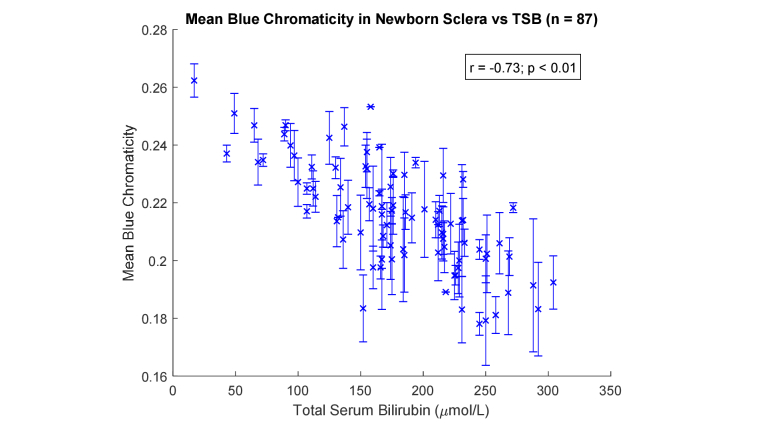
Mean blue chromaticity values versus total serum bilirubin in newborn sclera (n = 87).

Multiple images and regions of interest (e.g. sclera in both eyes) were considered when available. Figure 3

depicts the means and standard deviations for the blue chromaticity of 87 babies and the corresponding TSB. The correlation coefficient (r) between the mean blue chromaticity and TSB is −0.73 (p<0.01). The correlation is negative because a higher TSB would mean more blue light being absorbed and therefore a lower blue chromaticity. For comparison, the r between the mean blue values and TSB is −0.47 (p<0.01) (results not shown), substantially lower than that for the mean blue chromaticity.

### 3.2 CIE XYZ color space

Figure 4

**Fig. 4:**
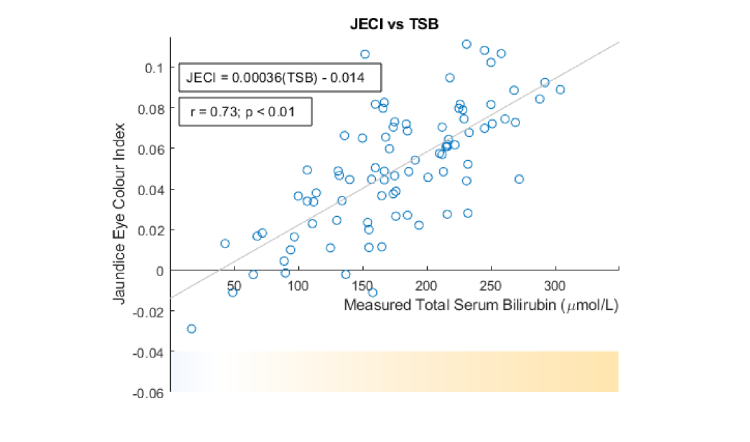
The Jaundice Eye Color Index (JECI) versus TSB. The horizontal color bar displays the yellowness of sclera corresponding to a specific TSB under D65 illuminant.

shows the scatter plot between TSB and JECI with r = 0.73 (p<0.01). A linear regression has also been performed, shown as the straight line in Fig. 4. The regressed line allows us to map TSB to JECI and therefore provides a “typical” scleral yellowness for a particular TSB in the neonatal population (under the D65 illuminant), as shown at the bottom of Fig. 4. In general, the higher the JECI, the more yellow the sclera. It is noted that JECI can also be negative, corresponding to a blueish color, which is often found in newborns with thin sclera. (A zero JECI corresponds to white under the D65 illuminant.)

## 4. Discussion and conclusion

In our previous publication, we used a multiple linear regression approach to find the relationship between the RGB values of the sclera and the TSB of the newborn babies [7]. The predicted TSB value resulting from the regression is a weighted sum of the RGB values, their squared values, and their cross-products. Due to a large number of parameters involved (9 in total), there is a risk of over-fitting the data, resulting in a technique which may only be accurate for one particular population. The regression approach is based on training data and therefore can be influenced by the TSB itself.

In this paper, we have taken a different approach. Instead of trying to find a model that can predict TSB directly, we investigate color metrics that can quantify the yellowness of the sclera. We have considered two training-free color metrics: blue chromaticity in the native RGB space and the newly-defined JECI in the CIE XYZ space. These metrics can be compared between different populations (e.g. term/pre-term neonates) even if the relationship between yellowness and TSB is not the same. In our data set, both blue chromaticity and JECI show strong correlation with TSB (−0.73 and 0.73 respectively; p<0.01 in both). While the native RGB space is defined relative to a specific camera, the advantage of JECI is that the CIE XYZ space is a reference color space containing all possible colors and is device-independent.

A previous study showed that conjunctival icterus was mainly found in jaundice babies with TSB>255 μmol/L, as determined by experienced clinicians [5]. Instead of a binary decision, our work has provided evidence that conjunctival icterus is a condition best characterized by a continuous grading scale, since TSB is a quantity that can vary continuously. To this end, we have proposed JECI as the grading scale to quantify the degree of conjunctival icterus (or the yellowness of the sclera). For instance, JECI = 0 corresponds to white sclera and JECI = 0.1 sclera with a high degree of yellowness. Interestingly, JECI can also be negative, e.g., JECI = −0.02 for blueish sclera often found in newborns.

One smartphone-based technique has used the scleral color to identify jaundiced adult patients with pancreatic cancer [8]. After considering 105 color features with different color spaces, color channels, pixel selection methods and RGB ratios, the study found that the ratio of green to blue channels in the RGB color space is one color metric that best correlates with the data. The green-to-blue ratio is similar to both blue chromaticity and JECI investigated in this paper in terms of their sensitivity to yellowness and normalization against the brightness of the scene. Our work provides further evidence that a normalized blue color metric correlates well with TSB, and a color-science-based justification for this.

One useful application of JECI is the regression of JECI against TSB to predict the “typical” yellowness of the sclera in a jaundiced newborn for a given TSB (Fig. 4). JECI can also be used to quantify the scleral yellowness in other jaundiced patient groups, such as adult liver and pancreatic patients. By applying the JECI metric to data sets from various jaundiced populations, researchers can help healthcare professionals visualize how scleral yellowness relates to TSB, and, if necessary, have this tailored for certain populations.

To quantify the yellowness of the sclera successfully, the color metric should fulfil the following requirements: (i) sensitive to yellowness (produces good contrast between yellows), (ii) normalized to reduce the effect of brightness in the scene, (iii) based on a device-independent color space, (iv) able to capture the full gamut of scleral yellowness, and (v) insensitive to ambient lightings. We are currently developing a diagnostic smartphone app based on JECI. To this end, BiliCam has already been developed to diagnose neonatal jaundice based on skin color [9]. Development of diagnostic camera apps is an exciting research area with many promising new cases being actively investigated, a trend that will only grow in the foreseeable future.
